# Management of Hereditary Hypofibrinogenemia During Pregnancy: A Scoping Review Towards Personalized Obstetric Care

**DOI:** 10.3390/jcm15124666

**Published:** 2026-06-16

**Authors:** Grigorios Karampas, Konstantinos Karkalemis, Anastasia Bagiasta, Dimitra Metallinou, Ermioni Tsarna, Marikaiti Lefaki, Chryssoula Staikou, Makarios Eleftheriades, Panagiotis Christopoulos, Marianna Politou

**Affiliations:** 1 Second Department of Obstetrics and Gynaecology, “Aretaieio” University Hospital, Medical School, National and Kapodistrian University of Athens, 11528 Athens, Greece; karampasgrig@gmail.com (G.K.); abagiasta@hotmail.com (A.B.); makarios@hotmail.co.uk (M.E.); dr_christopoulos@yahoo.gr (P.C.); 2 Department of Midwifery, School of Health and Care Sciences, University of West Attica, 12243 Athens, Greece; dmetallinou@uniwa.gr; 3 Laboratory of Heamatology, Blood Transfusion Unit, “Aretaieio” University Hospital, Medical School, National and Kapodistrian University of Athens, 11528 Athens, Greece; mklefaki@gmail.com (M.L.); mpolitou10@gmail.com (M.P.); 4 First Department of Anesthesiology, “Aretaieio” University Hospital, Medical School, National and Kapodistrian University of Athens, 11528 Athens, Greece; c_staikou@yahoo.gr

**Keywords:** hereditary hypofibrinogenemia, pregnancy, ROTEM^®^, FIBTEM^®^, perinatal outcome

## Abstract

**Background**: Hereditary fibrinogen disorders comprise a rare and heterogeneous group of conditions characterized by highly variable clinical phenotypes, ranging from entirely asymptomatic to severe hemorrhage or paradoxical thrombosis. Within this spectrum, hereditary hypofibrinogenemia (HH) poses a significant obstetrical challenge due to the lack of evidence-based management guidelines during pregnancy. **Methods**: A scoping review of the literature was conducted to identify reported cases of pregnancies with HH reaching the third trimester. PubMed, Scopus, and Cochrane Library were searched through April 2026 for eligible studies reporting maternal and neonatal outcomes, fibrinogen replacement therapy during pregnancy, and peri- and postpartum management. A complementary LeapSpace search was also performed. Data were extracted using a structured form and owing to the heterogeneity and descriptive nature of the available evidence, results were synthesized narratively. **Results**: Out of 202 unique records identified, a total of 13 studies, comprising 33 pregnancies, were included. All evidence arose from case reports and small case series, with substantial variability in patient characteristics and clinical management. Successful outcomes are associated with early diagnosis, careful assessment of medical and obstetrical history, and close multidisciplinary surveillance. Maintaining fibrinogen levels above 50–100 mg/dL during pregnancy and ≥150 mg/dL peripartum appeared beneficial. The use of global coagulation assessment tools such as rotational thromboelastometry (ROTEM^®^), particularly the FIBTEM^®^ assay, may support individualized management beyond fibrinogen levels alone; however, up to date it has been incorporated in the management of a single pregnancy. **Conclusions**: Management of pregnancy in women with HH should be individualized and multidisciplinary, with tailored fibrinogen supplementation strategies to optimize maternal and neonatal outcomes. Small sample sizes and the heterogeneity of the reported results limit the certainty of these findings, requiring further research to establish subtype-specific recommendations and to define additional coagulation parameters that may improve perinatal care.

## 1. Introduction

Fibrinogen is a 340-kD glycoprotein that is synthesized by parenchymal liver cells, has a half-life of four days and circulates in the blood stream in an average concentration of 150 to 350 mg/dL. It represents one of the core elements of the coagulation system facilitating the aggregation of activated platelets, while fibrin monomers, derived from fibrinogen after the activation of thrombin (FIIa), are crucial in fibrin clot formation [1]. Fibrinogen molecule consists of three paired polypeptide chains, originating from three different genes located on the chromosome 4, named alpha, beta and gamma, that form a hexamer [2]. Mutations to these three fibrinogen genes can lead to different disorders with distinct clinical presentation, collectively called hereditary fibrinogen disorders (HFDs) [3,4].

The HFDs represent a spectrum of uncommon and heterogeneous hereditary diseases, with largely unknown prevalence mainly due to their rarity and lack of symptoms in many cases [5]. The HFDs are further classified as quantitative, qualitative or a combination of both depending on the activity and the levels of fibrinogen. Quantitative fibrinogen disorders include afibrinogenemia, defined as complete lack of fibrinogen, and hypofibrinogenemia where fibrinogen level is low, further classified as mild (fibrinogen level more than 100 mg/dL), moderate (fibrinogen level between 50 and 90 mg/dL) and severe (fibrinogen level < 50 mg/dL) [5]. Qualitative HFDs are characterized by loss of fibrinogen function while maintaining normal (dysfibrinogenemia) or having low fibrinogen level (hypo-dysfibrinogenemia) [6].

Clinical manifestations of HFDs vary, ranging from asymptomatic presentations and self-limiting spontaneous bleeding—common in hypofibrinogenemia—to life-threatening hemorrhagic or thrombotic complications, which are more characteristic of afibrinogenemia and dysfibrinogenemia [5]. Women with HFDs represent a distinct patient group that is affected particularly during pregnancy and delivery. Fibrinogen plays a crucial role in the early stages of pregnancy, contributing to the establishment of the fetal–maternal vasculature [7]. It supports cytotrophoblast proliferation and subsequent invasion into the myometrium, processes which are fundamental to normal placental formation and function [7]. For this reason, women with HFDs are prone to various pregnancy complications including bleeding events, varying from recurrent pregnancy loss to placenta abruption and life-threatening postpartum hemorrhage (PPH) [8].

The management of different types of HFDs during pregnancy and peri-postpartum periods is primarily guided by expert consensus. Historically, replacement strategies relied on the administration of cryoprecipitate and fresh frozen plasma (FFP); however, these blood products present significant limitations, including risks to viral safety and potential volume overload. The expanding use of fibrinogen concentrates (FIBc) in the treatment of PPH has familiarized clinicians with their benefits, facilitating a transition toward the use of fibrinogen concentrates in HFD management as a prophylactic measure. This shift directly addressed the drawbacks associated with FFP and cryoprecipitate. Nevertheless, a remaining limitation was the lack of precision dosing, which stemmed from a reliance on static fibrinogen level rather than a holistic approach of the women’s coagulation system [6]. Recently, however, a novel individualized strategy for fibrinogen supplementation during pregnancy and peri-, intra-, and postpartum has been proposed. This approach utilizes the FIBTEM^®^ assay (a fibrin-based, extrinsically activated test with tissue factor and cytochalasin D) within the rotational thromboelastometry (ROTEM^®^) framework (Tem Innovations GmbH. ROTEM delta FIBTEM Reagent Package insert; Tem Innovations GmbH/Werfen: Munich, Germany). As a global test of hemostasis, ROTEM^®^ allows for more precise, real-time management of these rare high-risk pregnancies [9,10,11].

The objective of this scoping review was to systematically map and evaluate the existing literature regarding pregnancies complicated by hereditary hypofibrinogenemia (HH) that successfully progressed to the third trimester. In accordance with the PCC (Population, Concept, Context) framework for evidence synthesis, this study targeted a population of pregnant women with a clinical or genetic diagnosis of HH [11]. The concept examined encompasses obstetric management and fibrinogen replacement strategies, including clinical decision-making guided by traditional static fibrinogen measurements and/or the FIBTEM^®^ assay. These elements were evaluated within the context of third trimester pregnancies and their subsequent maternal and perinatal outcomes. Because current guidelines for managing HFDs during pregnancy are primarily based on expert consensus rather than high-level clinical evidence, this review aims to identify critical research gaps and synthesize available clinical data to better inform the development of evidence-based protocols.

## 2. Methods

To ensure a systematic selection of the relevant literature, studies were screened based on predefined eligibility criteria. Inclusion criteria comprised: (1) cases of clinically or genetically diagnosed HH during pregnancy; (2) pregnancies reaching the third trimester; (3) detailed reporting on treatment strategies; (4) publication in English or availability of essential data in English; and (5) studies published between January 1999 and December 2025. This timeframe was specifically selected to exclude evidence predating the widespread clinical use of fibrinogen concentrate (FIBc). Conversely, exclusion criteria included cases of secondary hypofibrinogenemia, afibrinogenemia (hereditary or secondary), dysfibrinogenemia, or combined hypo-dysfibrinogenemia. Due to the rarity of HH, all original study designs, primarily case reports and case series, were eligible, while reviews and editorials were excluded. Retrieved conference abstracts were excluded if they did not provide sufficient data to satisfy our concept (treatment strategies).

The search algorithm was initially developed for PubMed, using a combination of keywords and MeSH terms describing hypofibrinogenemia, pregnancy, and associated treatments (Table S1). This search algorithm was subsequently adapted for the Cochrane Library and Scopus using the Polyglot Search Translator (Table S1) [12]. The final search was updated on 22 April 2026. Additionally, a specialized prompt was used in LeapSpace, as a supplementary discovery layer to ensure the comprehensiveness of our primary database search (Table S1) [13]. Its purpose was to identify any case reports or series that might have been missed by traditional keyword/MeSH indexing. Specifically, the following prompt was used in LeapSpace: *“Can you identify case reports and case series of hereditary hypofibrinogenemia in pregnancy?”*. All studies identified via this strategy were screened for duplication against the primary databases’ search results.

All records identified from PubMed, Scopus, and Cochrane Library were introduced in Rayyan, where deduplication after manual confirmation was performed [14]. Two independent reviewers (K.K. and A.B. or E.T.) screened all titles and abstracts. Potentially relevant publications were screened in full text. In case of discrepancy, the first author (K.G.) reviewed the relative study to reach the final decision regarding inclusion. To avoid double-counting patients, study authors and institutions were cross-checked for overlapping cohorts.

Data extraction using a structured form was performed by the author K.K. and the accuracy was subsequently verified by the author G.K. The following information was extracted per study: first author’s name and year of publication, number of pregnancies per study, personal characteristics and medical history of each pregnant woman, including maternal age, initial diagnosis of the disorder, gravidity, parity, obstetric history, as well as obstetrical data such as week of labor, mode of delivery, neonate’s birth weight and APGAR score, method of analgesia or anesthesia received during delivery and ante-postpartum complications. Data about fibrinogen levels at different weeks of gestation, during labor and postpartum as well as postpartum antithrombotic therapy were also collected with focus on the fibrinogen supplementation treatment that was administered and, lastly, the treatments’ effect on the fibrinogen levels along with the occurrence of complications.

The data extracted from the reviewed studies were synthesized narratively, complemented by descriptive statistics for continuous variables, including the mean and standard deviation (SD). Potential sources of bias in the reviewed studies and certainty in the evidence were discussed in a group meeting among the authors, with the aim to identify key limitations in present knowledge and guide future research directions. In addition, the Joanna Briggs Institute (JBI) checklists for case series and case reports were used to perform a formal critical appraisal of the included studies [15,16]. The protocol for this review was not formally registered but will be made available upon request. This scoping review followed PRISMA extension for scoping reviews (PRISMA-ScR) (Table S2) [17].

## 3. Results

**Figure 1:**
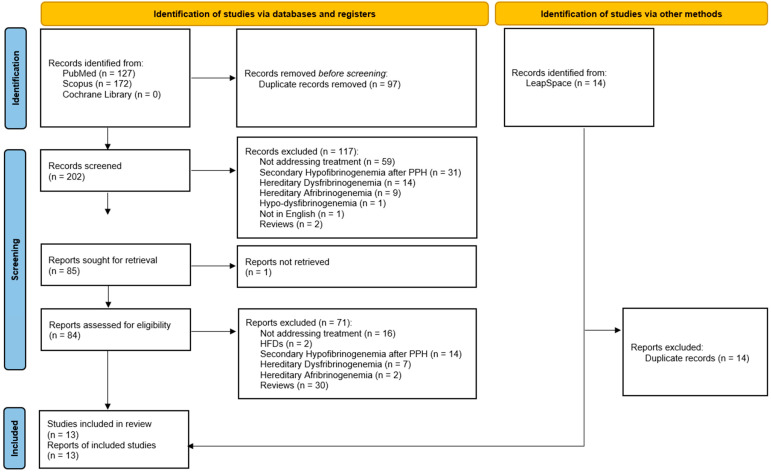
Flow diagram summarizing the study selection process for this scoping review.

Our search strategy, as presented in Figure 1, resulted in identification of 127 studies from PubMed and 172 from Scopus; no relevant studies were retrieved from Cochrane Library. Following the removal of 97 duplicates, 202 titles and abstracts were screened, resulting in the exclusion of 117 records. The primary reasons for exclusion at this stage included a lack of focus on treatment (*n* = 59), investigation of secondary hypofibrinogenemia following PPH (*n* = 31), or inclusion of other HFDs, such as dysfibrinogenemia (*n* = 14), afibrinogenemia (*n* = 9), or hypo-dysfibrinogenemia (*n* = 1). Further exclusions were based on non-English language (*n* = 1) and ineligible publication types (*n* = 2). Of the 85 reports sought for retrieval, one could not be obtained and it was presumably published in a language other than English. Consequently, 84 full-text articles were assessed for eligibility. Of these, 71 were excluded for the following reasons: treatment was not addressed (*n* = 16); subgroup data for HH patients were not reported separately from other HFDs (*n* = 2); other fibrinogen disorders were examined, including secondary hypofibrinogenemia (*n* = 14), hereditary dysfibrinogenemia (*n* = 7), and hereditary afibrinogenemia (*n* = 2); or the records were review articles (*n* = 30). All 14 studies identified from LeapSpace were already among the results of our primary databases search. The 100% overlap between the LeapSpace results and the primary database results served to validate the sensitivity of our original search algorithms. Ultimately, 13 studies met the criteria for inclusion in this scoping review [2,11,18,19,20,21,22,23,24,25,26,27,28].

Across 13 reviewed studies, 29 women with HH were identified accounting for a total of 33 singleton pregnancies resulting in live births. Three women contributed multiple pregnancies to the dataset: one woman was included for three separate pregnancies and two women were each included for two pregnancies occurring at different time points [2,11,18,19,20,21,22,23,24,25,26,27,28]. The case reported by Pietrzak et al. provided information only for maternal and neonatal characteristics and pregnancy outcomes, while no data on fibrinogen supplementation were reported [23].

Mean maternal age at first delivery was 32.56 ± 4.4 years and mean gestational age at labor for the first pregnancy was 38.6 ± 2.3 weeks (Table 1). The initial diagnosis of HH varied extensively; cases were identified due to family history, investigations for prior pregnancy complications, or were incidental findings during unrelated medical evaluations (Table 1). Regarding obstetric history, the most frequent complications reported were first trimester miscarriages (*n* = 6), placental abruption (*n* = 3), preterm delivery (*n* = 3), second trimester loss (*n* = 2) and severe PPH (*n* = 1). Notably, specific mutations were not reported in any of the included studies. Interestingly, fibrinogen supplementation during pregnancy was explicitly reported in only seven cases. In the majority of the remaining cases, no definitive mention of supplementation could be extracted. Conversely, in the study by Karampas et al. pregnancy management was guided by FIBTEM^®^ assay, which resulted in conservative follow-up without the requirement for supplementation [11].

**Figure 2:**
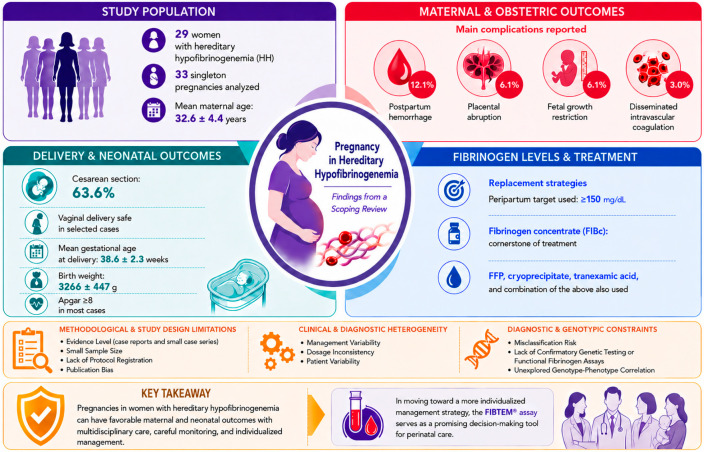
Summary of management strategies and clinical outcomes for hereditary hypofibrinogenemia in pregnancy: A scoping review mapping.

Cesarean section (CS) was the preferred mode of delivery in most cases (21/33 deliveries), including both elective and emergency procedures, with general anesthesia being the method most frequently used (Table 1). On the contrary, no data were available regarding the choice of analgesia method for vaginal deliveries. The mean birth weight was 3266.5 ± 447 g and first minute APGAR score was ≥8 in all cases with available data (Table 1). While most pregnancies proceeded without complications, the most common adverse event was PPH, either in isolation or in combination with placenta abruption. In one case, severe PPH necessitated hysterectomy and another case was complicated by disseminated intravascular coagulation (DIC) associated with placenta abruption (Table 1). Fetal growth restriction (FGR) was also identified as a recurring complication (*n* = 2) (Figure 2).

Fibrinogen levels during pregnancy exhibited significant variation, ranging from 22 mg/dL to 368 mg/dL, largely contingent upon the administration of supplementation therapy (Table 2). Antenatal supplementation included the use of either FIBc (*n* = 4) or cryoprecipitate (*n* = 3). Antepartum fibrinogen levels prior to the final dose of supplementation also varied widely, from undetectable levels in one instance to 311 mg/dL. Peripartum supplementation strategies were diverse, utilizing FIBc as monotherapy (*n* = 16), FIBc in combination with FFP (*n* = 6) or factor XIII (*n* = 1), tranexamic acid (*n* = 1), or cryoprecipitate as monotherapy (*n* = 1). Additionally, two cases required transfusion of whole blood and coagulation factors, while another two cases required comprehensive combination of FIBc, FFP, packed red blood cells (RBCs), platelets, and cryoprecipitate (Table 2).

The peripartum substitution dose of FIBc ranged extensively from 1 g to 12 g, depending on whether it was administered in isolation or as part of a multi-component therapy. In all cases where high FIBc dose was administered, the total dose was typically divided into smaller increments administered shortly before labor (ranging from three days to few hours antepartum). The mean antepartum fibrinogen level immediately preceding labor was 126.5 ± 71.3 mg/dL, while the intrapartum level (following treatment) rose to 165 ± 65.2 mg/dL (range 79–351 mg/dL). On the first postpartum day, the mean fibrinogen level was 153.9 ± 84.2 mg/dL (Table 2).

Postpartum antithrombotic therapy was explicitly detailed in only two studies by Casini et al. and Karampas et al., both of which utilized low-molecular-weight heparin (LMWH) [11,19].

Summary of the management strategies and clinical outcomes for hereditary hypofibrinogenemia in pregnancy are presented in detail in Figure 2.

Regarding the quality appraisal of the included literature, the case reports consistently provided clear accounts of the patients’ clinical conditions, therapeutic interventions, post-intervention outcomes, and adverse events, with each concluding with a takeaway message. However, minor omissions occurred; specific demographic data [19] or detailed clinical histories [23,28] were occasionally omitted. Notably, five of the eight case reports failed to clearly outline the exact diagnostic criteria or tests implemented to confirm the diagnosis of HH [18,19,23,26,28]. For the case series, all studies utilized clear inclusion criteria and transparently reported patient demographics, clinical characteristics, clinical outcomes, and presentation sites. However, several methodological limitations were identified: four studies demonstrated inconsistencies in applying standardized diagnostic protocols [27] or gold-standard diagnostic testing [2,20,21,27]. These same four studies also failed to demonstrate consecutive patient enrollment, with complete patient inclusion being lacking in two of them [2,27]. In conclusion, the primary sources of systematic error across the reviewed literature stem from potential selection bias and the misclassification of HH cases. It is important to note, however, that these deficiencies arose largely from omissions in documentation; consequently, it remains ambiguous whether these findings reflect true methodological flaws or incomplete reporting.

## 4. Discussion

**Principal Findings and Pathophysiology**

HH is typically an autosomal dominant condition, a fact supported by its equal prevalence in both sexes. Recent genetic insights from the Prospective Rare Bleeding Disorders Database (PRO-RBDD) indicate that HH is primarily driven by heterozygous missense or null mutations, predominantly within the fibrinogen gamma (FGG) gene [4]. While many women remain asymptomatic, the clinical phenotype is highly heterogeneous, ranging from minor epistaxis and cutaneous bleeding to severe menorrhagia, life-threatening bleedings, or paradoxical thromboses [8]. However, even in severe forms of HH, the diagnosis is often incidental or through evaluations unrelated to bleeding diathesis. The literature describes pregnancies that follow an uncomplicated course despite severe HH with fibrinogen levels consistently below 100 mg/dL, suggesting that clinical phenotype and individual bleeding history may be more reliable indicators for management than absolute fibrinogen concentrations alone [11].

Although HH does not typically impede conception and implantation, fibrinogen is a prerequisite for placental stabilization and establishment of maternal–fetal vasculature. Consequently, untreated or severe deficiency often leads to unfavorable outcomes, including miscarriages (typically between 5 and 8 weeks of gestation), FGR, preeclampsia, placental abruption, and severe PPH. These risks necessitate management within tertiary centers capable of close surveillance and immediate access to supplementation therapies [7].

Compared to other HFDs, HH appears to represent a more moderate risk profile. The evidence synthesized in this review suggests that when appropriately managed, HH is seldom associated with severe hemorrhagic or thrombotic complications once the third trimester is reached, with a favorable perinatal outcome observed in the majority of cases. In contrast, thrombotic events during pregnancy occur more frequently in cases of afibrinogenemia or dysfibrinogenemia; for these subtypes, the prophylactic use of low-molecular-weight heparin (LMWH) is considered a therapeutic alternative—an approach that is generally not indicated for HH [6]. These findings underscore the necessity for subtype-specific recommendations within the HFD spectrum during pregnancy, suggesting that an updated clinical consensus should address these distinct management requirements.

**Medical management during pregnancy**

Current expert consensus, such as the Delphi recommendations from the HFDs group, advocates for a dual-phase management strategy: the antenatal period and the intra–postpartum period [6,8]. Based on this expert consensus, which lacks high-level evidence, maintaining fibrinogen levels above 50–100 mg/dL during pregnancy to prevent pregnancy loss and exceeding 150 mg/dL peripartum to ensure adequate hemostasis during delivery is currently recommended [6]. Notably, the agreement between experts’ opinion regarding exact fibrinogen levels that may be considered safe during pregnancy and delivery was limited, reflecting the lack of data regarding obstetric management of HFDs. In the referenced Delphi consensus, the administration of LMWH for postpartum thromboprophylaxis was recommended only in the presence of additional clinical indications, rather than as a routine measure for all patients with HFDs [6].

Current guidelines recommend vigilant monitoring of fibrinogen levels throughout gestation [6,8]. In clinical practice, this typically involves monthly assessments conducted by an experienced hematologist [6]. Notably, the specific subtype of HFD—namely afibrinogenemia, hypofibrinogenemia, or dysfibrinogenemia—alongside the severity of symptoms in the patient’s personal and family medical history, serves as a critical framework for personalizing treatment strategies during pregnancy and delivery [6].

To achieve targeted fibrinogen levels, three primary replacement therapies are available, namely fresh frozen plasma (FFP), cryoprecipitate, and FIBc, with FIBc currently regarded as the optimal therapeutic choice [29,30]. Unlike FIBc, both cryoprecipitate and FFP necessitate ABO compatibility and result in the administration of extraneous coagulation factors, such as factor VIII and von Willebrand factor, which may be unnecessary for the patient. Furthermore, the use of FFP and cryoprecipitate carries inherent risks of pathogen transmission and allergic reactions due to the potential presence of anaphylatoxins or blood-borne pathogens [30,31]. FIBc is typically provided in a purified 50 mL volume at a concentration of 20 mg/mL, totaling 1 g per unit. Clinically, one unit of FIBc is expected to increase plasma fibrinogen levels by approximately 30 mg/dL. This pharmacological profile underscores why most obstetric cases of hypofibrinogenemia require a peripartum administration of at least 3 g of FIBc to reliably secure fibrinogen levels >150 mg/dL. Nevertheless, in certain cases very high doses of FIBc are required peripartum to achieve target plasma fibrinogen levels of 100–150 mg/dL; however, the impact of such aggressive replacement on a patient’s thrombotic risk remains poorly understood [11,32,33]. The administration of escalated intravenous doses warrants caution, as it may increase the risk of thromboembolic events without providing a proportional benefit to hemostasis—particularly when global hemostatic parameters, such as those measured by the FIBTEM^®^ assay, remain within normal limits [11].

The historical reliance on static fibrinogen levels as the primary determinant of HH therapy during pregnancy may overlook critical functional aspects of the coagulation system. This limitation is underscored by the study of Karampas et al., which presented two distinct therapeutic strategies applied to the same woman across two pregnancies [11]. In the first pregnancy, a traditional approach utilized bi-weekly supplementation of 2 g of FIBc to maintain levels ≥100 mg/dL, alongside a 6 g peripartum dose. Conversely, the second pregnancy utilized a conservative strategy guided by the ROTEM^®^ global hemostasis test—specifically the FIBTEM^®^ assay—which resulted in the omission of antenatal supplementation and the use of a divided 6.5 g peripartum FIBc dose. Both pregnancies yielded favorable perinatal outcomes, demonstrating that a holistic, individualized assessment of hemostasis may prove helpful in minimizing unnecessary clinical intervention.

In moving toward a more individualized management strategy, FIBTEM^®^ assay, already established in perioperative fibrinogen replacement in patients with HFDs, offers useful insights and a more holistic evaluation of maternal coagulation status [11,34]. Its utility is particularly significant in assessing functional clot elasticity, because complex interactions of fibrinogen with other coagulation factors, including Factor XIII, are reflected in parameters such as clot amplitude 10 min after clotting time (A10) and maximum clot firmness (MCF) [34,35,36]. By providing a more comprehensive view of a patient’s coagulation status and homeostasis compared with concentration measurements alone, viscoelastic parameters like FIBTEM^®^-MCF serve as promising decision-making tools for individualized perinatal care that may contribute in reducing both unnecessary interventions during pregnancy and thrombotic risks peripartum [34,35,36]. In addition, FIBTEM^®^ assay may facilitate the differential diagnosis of HFD subtypes when they are first identified during pregnancy. Pregnancy-induced alterations in multiple coagulation factors often complicate the diagnosis of hemostatic disorders [1,37,38]. In HH, abnormal FIBTEM^®^ parameters are unlikely to be present, whereas in dysfibrinogenemia, these parameters are typically ambiguous or overtly pathological [39,40].

**Optimal management of delivery and labor**

The attainment of an uncomplicated perinatal outcome in cases of HH relies heavily on rigorous labor management, encompassing the mode of delivery, fetal surveillance techniques, and the selection of analgesia or anesthesia. Given the predominantly autosomal dominant inheritance of HH, every fetus must be regarded as being at high risk for hemorrhagic complications. Neonates with the combination of expected developmental hemostatic immaturity and the presence of inherited coagulation disorders are associated with significant risks of both hemorrhagic and thrombotic complications. Intraventricular hemorrhage (IVH), various neonatal extracranial or intracranial injuries, such as cephalohematoma, subgaleal hemorrhage, subdural hemorrhage, and subarachnoid hemorrhage, and umbilical cord hemorrhage can occur during both vaginal delivery and CS [7,8].

Hypofibrinogenemia in the mother or the fetus can manifest as an acute obstetric emergency, including placental abruption, antepartum hemorrhage, or non-reassuring fetal status, often necessitating immediate delivery. In the studies analyzed in this scoping review, no severe neonatal complications were reported; however, PPH occurred in four of the 33 cases (12.1%). Emergency cesarean sections and intensive PPH management were occasionally required. Notably, another review of HH in pregnancy—which included untreated patients—reported PPH in 10 out of 37 cases (27.0%), underscoring the significantly elevated risks associated with less rigorous clinical monitoring [8]. Nonetheless, these comparisons are descriptive in nature and subject to significant publication bias and should not be interpreted as a statistical claim of effectiveness. Notably, neonatal risks are not expected to be entirely mitigated even in well-monitored and treated women with HH; since fibrinogen cannot cross the placental barrier due to its large molecular weight, the fetal coagulation status remains independent of maternal supplementation.

Our findings suggest that both vaginal delivery and CS are viable options for women with HH, provided appropriate precautions are maintained. However, vaginal delivery should be strictly supervised by a senior obstetrician, as operative interventions involving forceps or vacuum extraction significantly increase the risk of maternal and neonatal hemorrhagic complications [41,42]. Furthermore, the use of invasive fetal monitoring—including internal cardiotocography (scalp electrodes), fetal scalp blood sampling (FBS), or ST-waveform analysis (STAN)—is generally contraindicated or should be avoided due to the undetermined risk of iatrogenic fetal hemorrhage [43,44,45]. Consequently, an individualized labor management plan should integrate maternal obstetric history (parity and gravidity), physical characteristics (height, weight, and BMI), and current pregnancy parameters, such as ultrasound-estimated fetal weight and comorbid pathologies like diabetes mellitus [46,47,48].

The selected delivery mode significantly influences the choice of analgesia or anesthesia [49]. Generally, central neuraxial blocks are contraindicated in patients with untreated coagulation disorders due to the elevated risk of spinal or epidural hematoma [49]. For this reason, general anesthesia is typically the preferred modality for CS in patients with HH [50]. Nevertheless, regional anesthesia has been successfully utilized in select cases where the coagulation defect was sufficiently corrected (e.g., fibrinogen levels reaching approximately 300 mg/dL) [51]. Current guidelines suggest that in elective scenarios, regional techniques may be considered only after a satisfactory response to replacement therapy and clinical clearance by a hematologist [49]. For vaginal deliveries where neuraxial techniques are deemed unsafe, alternative analgesia—including pudendal or paracervical blocks, local infiltration, nitrous oxide, parenteral opioids (e.g., remifentanil), non-opioid analgesics, sterile water injections, transcutaneous electrical nerve stimulation, acupuncture, relaxation (e.g., yoga) and manual techniques (e.g., massage)—should be considered either in isolation or in combination to ensure maternal comfort [52].

**Limitations**

This scoping review is subject to several limitations inherent to the study of rare diseases. The available literature consists of case reports and small case series, which are typically excluded from systematic reviews of interventions, due to the lack of randomization, blinding, and controls, and there is well-known high potential for publication bias, where successful or extraordinary outcomes are more likely to be reported than adverse or common ones [53]. Furthermore, there is substantial heterogeneity in clinical management across different centers, particularly regarding the threshold for and type of fibrinogen supplementation used. Importantly, dosage heterogeneity exists, even when similar thresholds and similar supplementation are utilized. The source of this heterogeneity may be attributed to genetic variability and individualistic characteristics of patients, attributing also to selection bias in such studies [53]. However, the potential of misclassification of diagnosis should also be considered, due to the use of a pragmatic inclusion threshold of author-diagnosed HH in our scoping review methodology. In seven out of 29 cases, HH diagnosis was declared without providing any details to ensure that concurrent dysfibrinogenemia was excluded. Genetic testing was reported only in two cases, while functional fibrinogen tests were reported in five cases. In addition, the lack of genetic mutation data prevents a correlation analysis between genotype and clinical phenotype. Furthermore, the protocol for this review was not prospectively registered in a public database. While this may be viewed as a limitation regarding formal transparency, the review followed a predefined internal protocol to minimize the risk of selective reporting. Methodological refinements, such as the expansion of the search to additional databases beyond PubMed and the inclusion of a formal critical appraisal, were implemented to ensure a more comprehensive evidence synthesis and to enhance the rigor of the reported findings. Specifically, the methodological quality of the included case reports and case series was assessed using the Joanna Briggs Institute (JBI) critical appraisal checklists, as presented in Tables S3 and S4. The aforementioned limitations coupled with the observed small sample sizes in the reviewed case series prevent the extrapolation of results into clinical practice.

**Future Research Directions**

The findings of this review highlight several critical gaps in our understanding of HH in pregnancy that warrant further investigation. Future research must clarify whether different HFD subtypes, such as HH versus dysfibrinogenemia, represent indeed divergent risk profiles for thrombosis and hemorrhage, thereby facilitating the development of subtype-specific anticoagulation and supplementation guidelines [1,6]. In addition, research should prioritize the exploration of genotype–phenotype correlations among HH patients by identifying specific fibrinogen gene mutations to determine if distinct variants require different thresholds for clinical intervention. The extent to which a woman’s personal, family, and obstetric history should dictate therapeutic choices is yet to be determined. Additionally, prospective studies are necessary to validate the use of FIBTEM^®^-guided protocols against standard-of-care static fibrinogen targets; such validation is crucial for establishing viscoelastic assay safety in reducing the volume of FIBc administered. Ultimately, further evidence is needed to identify the most suitable supplementation options and to define the optimal therapeutic and labor management strategies—including delivery methods, anesthesia choices, and neonatal support—for these high-risk pregnancies. Lastly, systematic follow-up of neonates born to mothers with HH is required to establish long-term safety data and determine the incidence of neonatal hemorrhagic complications. Given the rarity of HH in the obstetric population, addressing these knowledge gaps necessitates the establishment of an international study group. Such a collaboration would facilitate adequate study enrollment and the prospective collection of harmonized data, ensuring that maternal and neonatal monitoring accounts for all theoretical and empirically proven risks associated with HH.

## 5. Conclusions

HH is a rare disease, representing an obstetrical challenge. A multidisciplinary approach, involving minimally a hematologist and a senior obstetrician, and a care plan tailored to the specific needs of each patient are of utmost importance. Current evidence mapping suggests that management often focuses on early diagnosis, information gained from the medical-obstetrical history, close surveillance, and maintenance of fibrinogen levels above 50–100 mg/dL throughout pregnancy and at least ≥150 mg/dL peripartum. Fetal growth should be monitored during pregnancy by ultrasound and fetal wellbeing by continuous cardiotocography during labor, in cases of vaginal delivery. In the future, genotype–phenotype correlations and the addition of specific parameters of the ROTEM^®^ global test of hemostasis such as FIBTEM^®^ assay should be studied, as they may contribute towards a more individualized perinatal care assuring optimal outcomes.

## Figures and Tables

**Table 1:** Summary of findings table regarding maternal and obstetric characteristics and perinatal outcome.

Study	Case (Gravidity and Parity)	Age (Years)	Initial Diagnosis	Obstetric History	Gestational Age at Delivery	Mode of Delivery	Anesthesia/Analgesia	Birth Weight (g)	APGAR Score (1st/5th min)	Complications
Frenkel et al. 2004 [21]	1 (G4P2)	37	Incidental finding at 19 weeks of the 3rd pregnancy	PPH	-	VD	-	-	-	-
	2a (G1P0)	25	At work up for placental abruption & thrombophilia	-	36 w	ECS	-	2200	-	Placenta abruption —DIC
	2b (G2P1)	-	-	-	30 w	ECS	-	-	-	Placental abruption—severe PPH
	2c (G3P2)	-	-	-	35 w	CS	-	-	-	FGR
Hanke AA et al. 2010 [24]	1 (G1P0)	32	-		38 w	CS (breech presentation)	Spinal analgesia	-	-	-
Kaparou et al. 2012 [26]	1 (G3P1)	34	Work up due to positive family history	Abortion due to placenta abruption at 10 w (CS) & transfusion with 3 RBCs	39 w	CS (previous CS)	-	3050	-	-
Teraoka et al. 2017 [27]	1 (G2P1)	26	Incidental finding during bone marrow donor work up	First labor at 39 weeks delivery without fibrinogen substitution	38 + 2 w	VD	-	3206	-	828 mL blood loss
	2b (G3P1)	30	After emergency CS in the 1st pregnancy	Spontaneous abortion & placental abruption at 40 + 2 w	36 + 2 w	CS	GA	1724	8′ 10″	FGR diagnosis at 27 w, hospitalization since 31 w
Cai et al. 2018 [22]	1	-	Work up due to positive family history (8/11)	-	39 + 1 w	CS	-	3600	10′ 10″	-
	2	-		-	40 + 2 w	CS	-	3780	10′ 10″	-
	3	-		-	39 + 1 w	VD	-	3510	10′ 10″	-
	4a	-		-	40 + 1 w	FD	-	3530	10′ 10″	-
	4b	-		-	38 + 3 w	VD	-	3000	10′ 10″	-
	5	-		-	40 + 2 w	VD	-	3620	10′ 10″	-
	6	-		-	39 + 3 w	VD	-	3020	10′ 10″	-
	7	-		-	39 w	VD	-	3110	10′ 10″	-
	8	-		-	38 + 5 w	VD	-	3060	10′ 10″	-
	9	-		-	39 + 2 w	CS	-	3370	10′ 10″	-
	10	-		-	38 w	CS	-	3200	10′ 10″	-
	11	-		-	40 + 2 w	VD	-	3390	10′ 10″	-
Casini 2025 [19]	1	26	-	-	39 w	VD	Neuraxial anesthesia	-	-	Placental abruption at 2nd trimester
Li CQL et al. 2018 [20]	1 (G3P1)	34	-	-	37 + 6 w	CS	CSEA	-	-	-
	2 (G2P1)	34	-	-	38 + 6 w	CS	CSEA	-	-	-
	3 (G8P1)	40	-	Six 1st trimester miscarriages	37 w	CS	Single subaranchnoid block	-	-	-
	4 (G1P0)	33	-	-	37 w	CS	CSEA	-	-	-
Watts Soares et al. 2020 [28]	1	27	-	-	Full-term	VD	-	-	-	-
Pietrzak et al. 2014 [23]	1 (G2P1)	38	After CS due to TRALI & subfascial hematoma	One miscarriage	40 w	CS	-	2840	-/10″	600 mL blood loss—relaparotomy due to subfascial hematoma & anemia
Winebrenner et al. 2021 [18]	1 (G3P2)	38	-	History of placenta previa (vaginal bleeding at 24 w)	30+ 6 w	ECS	GA	-	8′ 9″	Placenta previa/bleeding (965 mL blood loss)
Li S et al. 2023 [2]	1 (G3P1)	30	-	One 1st trimester miscarriage		CS	GA	3300	10′ 10″	PPH—causing collapse and requiring hysterectomy
	2 (G2P1)	35	-	One 1st trimester miscarriage	38 + 3 w	CS	GA	3300	8′ 10″	-
Xie et al. 2025 [25]	1 (G1P1)	32	Work up due to need for long duration of compression at the venipuncture site and family history of PPH	Two chemical pregnancy and one early pregnancy loss	38 + 4 w	CS	-	-	-	-
Karampas et. al. 2025 [11]	1a (G3P0)	35	During investigation for two 1st trimester miscarriages	Two 1st trimester miscarriages	40 + 1 w	CS	GA	3350	9′ 10″	-
	1b (G4P1)	37	-	-	38 + 1 w	Repeat CS	GA	3370	9′ 10″	-
Mean ± SD	-	32.8 ±4.4	-	-	37 + 6 w ± 3 + 2	-	-	3266.5 ± 447	-	-

CSEA: combined spinal epidural analgesia, CS: cesarean section, DIC: disseminated intravascular coagulation, ECS: elective cesarean section, FGR: fetal growth restriction, GA: general anesthesia, PPH: postpartum hemorrhage, RBC: red blood cell, VD: vaginal delivery, TRALI: transfusion-related acute lung injury.

**Table 2:** Summary of findings table regarding fibrinogen levels during pregnancy, immediately antepartum, intrapartum, and at first postpartum day, along with supplementation therapy administered.

Study	Case	Fibrinogen Levels During Pregnancy (mg/dL)	Supplementation Therapy During Pregnancy	Fibrinogen Levels Immediately Antepartum (mg/dL)	Peripartum Therapy	Fibrinogen Levels Intrapartum (mg/dL)	Fibrinogen Levels 1st Day Postpartum (mg/dL)
Frenkel et al. 2004 [21]	1	Before diagnosis—19 w: 79 After diagnosis cryopercipitate treatment for amniocentesis: 198 Target pregnancy levels after diagnosis: 119–139	Cryopercipitate treatment before amniocentesis	136	10 units cryopercipitate	-	-
	2a	26 w: 134	-		Transfusion of blood and coagulation factors due to DIC	-	-
	2b	Early 1st trimester: 118	-	Undetectable before CS	Transfusion of blood and coagulation factors due to severe PPH and DIC	-	-
	2c	27 w: 98	10 units cryopercipitate weekly from 27th to 35th week (target fibrinogen levels > 250 mg/dL)			-	-
Hanke AA et al. 2010 [24]	1	-	-	104	4 g FIBc and 1250 unit of factor XIII	193	209
Kaparou et al. 2012 [26]	1	-	-	78	9 g FIBc	144	-
Teraoka et al. 2017 [27]	1	1st trimester: 119 3rd trimester: 300	-	311	-	-	-
	2	6 w: 75.9	3 g of FIBc weekly and every 3rd week at 3rd trimester (target > 100 mg/dL)	100	3 g FIBc for 3 consecutive days, 2 days before operation to maintain >200 mg/dL	228	281
Cai H et al. 2018 [22]	1	43–83	-	57	6 g FIBc	168	118
	2	67–100	-	99	4 g FIBc & 400 mL FFP	189	138
	3	52–61	-	60	2 g FIBc	79	67
	4a	48–80	-	75	3 g FIBc & 400 mL FFP	141	105
	4b	50–60	-	60	4 g FIBc & 400 mL FFP	123	90
	5	140–148	-	140	FFP 400 mL	254	199
	6	70–85	-	80	2 g FIBc	156	254
	7	70–96	-	95	2 g FIBc	140	123
	8	22–48	-	37	4 g FIBc & 400 mL FFP	142	58
	9	87–124	-	94	2 g FIBc & 400 mL FFP	163	66
	10	95–111	-	109	2 g FIBc	156	104
	11	65–95	-	73	2 g FIBc & 400 mL FFP	124	103
Casini 2025 [19]	1	-	FIBc supplementation 1st trimester (from 5th week): 50mg/kg × 2/week 2nd trimester: 50mg/kg × 3/week 3rd trimester: 75mg/kg × 3/week	150	1.5 g tranexamic acid IV	-	-
Li CQL et al. 2018 [20]	1	66–73	8 g FIBc in 3rd trimester	126	2 g FIBc 18 h before CS, 2 g FIBc 3 h after CS	131	-
	2	91–146	-	167	2 g FIBc 21 h and 2 g 6 h before CS, 2 g FIBc intraoperatively	163	-
	3	83–125	-	250	2 g FIBc 17 h and 2 g 3 h before CS, 2 g FIBc intraoperatively, 2 g FIBc 0.5 h after CS	321	-
	4	81–88	-	268	2 g FIBc 48 h and 2 g 24 h before CS, 4 g FIBc intraoperatively, 2 g FIBc 2 h after CS	351	-
Watts Soares et al. 2020 [28]	1	-	-	-	1 g FIBc preoperatively	-	-
Winebrenner et al. 2021 [18]	1	24 w: 85 27 w before treatment: <60 27 w after treatment: 158 S	4 doses of cryopercipitate at 27 + 2 weeks	-	5.7 g FIBc	150	-
Li S et al. 2023 [2]	1	3 months before delivery: 368	-	180	1 g FIBc preoperatively and 4 RBC, 4FFP, 40 units cryopercipitate perioperatively	126	-
	2	33 + 3 w: 353 38 + 3 w: 254	-	214	12 g FIBc, 1RBC, 1 platelets, 650 mL plasma, 20 IU/mL heparin	123	336
Xie et al. 2025 [25]	1	66–117	2 g FIBc at egg retrieval (before pregnancy)	77	6 g FIBc prior to CS in 3 doses	213	211
Karampas et. al. 2025 [11]	1a	-	2 g FIBc every two weeks to delivery	-	6 g FIBc at 40 + 0 prior to the elective CS	-	-
	1b	1st trimester: 69.1 2nd trimester: 58–84 3rd trimester: 67.6–82.3	No supplementation	88	1.5 g FIBc at 37 + 1, 1.5 g FIBc at 37 + 6 and 3 g FIBc at 38 weeks. Perioperatively 1 g tranexamic acid. Intraoperatively 1 g FIBc and 1 unit of FFP	97	-

CS: cesarean section, DIC: disseminated intravascular coagulation, FFP: fresh frozen plasma, FIBc: fibrinogen concentrate, RBC: red blood cell.

## Data Availability

The original contributions presented in this study are included in the article/Supplementary Material. Further inquiries can be directed to the corresponding author.

## Supplementary Materials

The following supporting information can be downloaded at: https://www.mdpi.com/article/10.3390/jcm15124666/s1, Table S1: Search algorithm in PubMed, Scopus, and Cochrane Library, and the Prompt utilized in LeapSpace. Table S2: PRISMA-ScR Checklist, Table S3: Quality Appraisal of the Included Case Series Studies with the Joanna Briggs Institute (JBI) checklists for Case Series, Table S4: Quality Appraisal of the Included Case Series Studies with the Joanna Briggs Institute (JBI) checklists for Case Reports.

## Abbreviations

The following abbreviations are used in this manuscript:

HFDs	Hereditary fibrinogen disorders
HH	Hereditary hypofibrinogenemia
SHH	Severe hereditary hypofibrinogenemia
ROTEM^®^	Rotational thromboelastometry
FIBTEM^®^	Fibrin-based thromboelastometry
CS	Cesarean section
IV	Intravenously
FIBc	Fibrinogen concentrate
MCF	Maximum clot firmness
FGR	Fetal growth restriction
FFP	Fresh frozen plasma
LMWH	Low-molecular-weight heparin
PPH	Postpartum hemorrhage
