# Supplementary material for: Management of Hereditary Hypofibrinogenemia During Pregnancy: A Scoping Review Towards Personalized Obstetric Care
Source: J Clin Med. 2026 Jun 16;15(12):4666. doi: 10.3390/jcm15124666 (PMC13302485; doi:10.3390/jcm15124666)
Supplement: Supplementary file 1 [file jcm-15-04666-s001.zip › jcm-4218928-supplementary.pdf]

Databases & other Sources	Search Algorithm or Prompt
**PubMed**	(("hypofibrinogenaemia"[All Fields] OR "afibrinogenemia"[MeSH Terms] OR "afibrinogenemia"[All Fields] OR "hypofibrinogenemia"[All Fields]) AND ("pregnancy"[MeSH Terms] OR "pregnancy"[All Fields] OR "pregnancies"[All Fields]) AND ("therapeutics"[MeSH Terms] OR "therapeutics"[All Fields] OR "treatments"[All Fields] OR "therapy"[MeSH Subheading] OR "therapy"[All Fields] OR "treatment"[All Fields] OR "treatments"[All Fields])) AND (1999:2025[pdat])
**Scopus**	((hypofibrinogenaemia OR afibrinogenemia OR afibrinogenemia OR hypofibrinogenemia) AND (pregnancy OR pregnancy OR pregnancies) AND (therapeutics OR therapeutics OR treatments OR therapy OR therapy OR treatment OR treatments))
**Cochrane Library**	((hypofibrinogenaemia OR [mh afibrinogenemia] OR afibrinogenemia OR hypofibrinogenemia) AND ([mh pregnancy] OR pregnancy OR pregnancies) AND ([mh therapeutics] OR therapeutics OR treatments OR [mh therapy] OR therapy OR treatment)) AND (1999:2025[pdat])
**LeapSpace**	“Can you identify case reports and case series of Hereditary hypofibrinogenemia in pregnancy ?”

Supplementary Table S1: The search algorithm in PubMed, Scopus, and Cochrane Library, and the Promp t utilized in LeapSpace.

SECTION	ITEM	PRISMA-ScR CHECKLIST ITEM	REPORTED ON
**TITLE**			
Title	1	Identify the report as a scoping review.	Title
**ABSTRACT**			
Structured summary	2	Provide a structured summary that includes (as applicable): background, objectives, eligibility criteria, sources of evidence, charting methods, results, and conclusions that relate to the review questions and objectives.	Abstract
**INTRODUCTION**			
Rationale	3	Describe the rationale for the review in the context of what is already known. Explain why the review questions/objectives lend themselves to a scoping review approach.	Introduction: 4th prg
Objectives	4	Provide an explicit statement of the questions and objectives being addressed with reference to their key elements (e.g., population or participants, concepts, and context) or other relevant key elements used to conceptualize the review questions and/or objectives.	Introduction: 5th prg
**METHODS**			
Protocol and registration	5	Indicate whether a review protocol exists; state if and where it can be accessed (e.g., a Web address); and if available, provide registration information, including the registration number.	Methods: 5th prg
Eligibility criteria	6	Specify characteristics of the sources of evidence used as eligibility criteria (e.g., years considered, language, and publication status), and provide a rationale.	Methods: 1st prg
Information sources*	7	Describe all information sources in the search (e.g., databases with dates of coverage and contact with authors to identify additional sources), as well as the date the most recent search was executed.	Methods: 2nd prg
Search	8	Present the full electronic search strategy for at least 1 database, including any limits used, such that it could be repeated.	Supplementary Table 1
Selection of sources of evidence	9	State the process for selecting sources of evidence (i.e., screening and eligibility) included in the scoping review.	Methods: 3rd prg
Data charting process	10	Describe the methods of charting data from the included sources of evidence (e.g., calibrated forms or forms that have been tested by the team before their use, and whether data charting was done independently or in duplicate) and any processes for obtaining and confirming data from investigators.	Methods: 4th prg
Data items	11	List and define all variables for which data were sought and any assumptions and simplifications made.	Methods: 4th prg
Critical appraisal of individual	12	If done, provide a rationale for conducting a critical appraisal of included sources of	Methods: 5th prg

SECTION	ITEM	PRISMA-ScR CHECKLIST ITEM	REPORTED ON
sources of evidence		evidence; describe the methods used and how this information was used in any data synthesis (if appropriate).	
Synthesis of results	13	Describe the methods of handling and summarizing the data that were charted.	Methods: 5th prg
**RESULTS**			
Selection of sources of evidence	14	Give numbers of sources of evidence screened, assessed for eligibility, and included in the review, with reasons for exclusions at each stage, ideally using a flow diagram.	Results: 1st prg<br>Figure 1
Characteristics of sources of evidence	15	For each source of evidence, present characteristics for which data were charted and provide the citations.	Results: 2nd prg
Critical appraisal within sources of evidence	16	If done, present data on critical appraisal of included sources of evidence (see item 12).	Discussion: Limitations Section
Results of individual sources of evidence	17	For each included source of evidence, present the relevant data that were charted that relate to the review questions and objectives.	Tables 1 & 2
Synthesis of results	18	Summarize and/or present the charting results as they relate to the review questions and objectives.	Results: 3rd – 7th prg
**DISCUSSION**			
Summary of evidence	19	Summarize the main results (including an overview of concepts, themes, and types of evidence available), link to the review questions and objectives, and consider the relevance to key groups.	Discussion:<br>Principal Findings and Pathophysiology 3rd prg, Medical management during pregnancy 4th and 5th prg, Optimal management of delivery and labor 2nd prg, Future Research Directions
Limitations	20	Discuss the limitations of the scoping review process.	Discussion: Limitations Section
Conclusions	21	Provide a general interpretation of the results with respect to the review questions and objectives, as well as potential implications and/or next steps.	Conclusion
**FUNDING**			
Funding	22	Describe sources of funding for the included sources of evidence, as well as sources of funding for the scoping review. Describe the role of the funders of the scoping review.	Funding Statement

Supplementary Table S2: Preferred Reporting Items for Systematic reviews and Meta-Analyses extension for Scopi ng Reviews (PRISMA-ScR) Checklist.

	Cai et al. 2018	Li CQL et al. 2018	Frenkel et al. 2004	Li S. et al. 2023	Teraoka et al. 2017
Were there clear criteria for inclusion in the case series?	YES	YES	YES	YES	YES
Was the condition measured in a standard, reliable way for all participants included in the case series?	YES	YES	YES	YES	NO
Were valid methods used for identification of the condition for all participants included in the case series?	YES	UNCLEAR	UNCLEAR	NO	UNCLEAR
Did the case series have consecutive inclusion of participants?	YES	UNCLEAR	UNCLEAR	UNCLEAR	UNCLEAR
Did the case series have complete inclusion of participants?	YES	YES	YES	NO	NO
Was there clear reporting of the demographics of the participants in the study?	YES	YES	YES	YES	YES
Was there clear reporting of clinical information of the participants?	YES	YES	YES	YES	YES
Were the outcomes or follow up results of cases clearly reported?	YES	YES	YES	YES	YES
Was there clear reporting of the presenting site(s)/clinic(s) demographic information?	YES	YES	YES	YES	YES
Was statistical analysis appropriate?	YES	YES	NA	NA	NA

Supplementary Table S3: Quality Appraisal of the Included Case Series Studies with the Joanna Briggs Institute (JBI) checklists for Case Series

	Kaparou et al. 2012	Watts Soares et al. 2020	Xie et al. 2025	Karampas et al. 2025	Hanke et al. 2010	Winebrenner et al. 2021	Casini et al. 2025	Pietrzak et al. 2014
Were patient’s demographic characteristics clearly described?	YES	YES	YES	YES	YES	YES	NO	YES
Was the patient’s history clearly described and presented as a timeline?	YES	NO	YES	YES	YES	YES	YES	Unclear
Was the current clinical condition of the patient on presentation clearly described?	YES	YES	YES	YES	YES	YES	YES	YES
Were diagnostic tests or assessment methods and the results clearly described?	Unclear	Unclear	YES	YES	YES	Unclear	Unclear	Unclear
Was the intervention(s) or treatment procedure(s) clearly described?	YES	YES	YES	YES	YES	YES	YES	YES
Was the post-intervention clinical condition clearly described?	YES	YES	YES	YES	YES	YES	YES	YES
Were adverse events (harms) or unanticipated events identified and described?	YES	YES	YES	YES	YES	YES	YES	YES
Does the case report provide takeaway lessons?	YES	YES	YES	YES	YES	YES	YES	YES
Overall appraisal	Include	Include	Include	Include	Include	Include	Include	Include

Supplementary Table S4: Quality Appraisal of the Included Case Series Studies with the Joanna Briggs Institute (JBI) checklists for Case Reports
